# Major changes in Pharmacognosy Magazine

**DOI:** 10.4103/0973-1296.71780

**Published:** 2010

**Authors:** K. K. Mueen Ahmed

**Affiliations:** *Assistant Professor, College of Clinical Pharmacy, King Faisal University, P. O. Box 400, Al-Ahsa-31982, Kingdom of Saudi Arabia*

Since 2005, Pharmacognosy Magazine (Phcog Mag) [www.phcog.com] is being published every quarterly without interruption. It serves the need of different scientists and others involved in medicinal plant research and development. Phcog Mag is independently grown and structured without any affiliations or any organizational support. We have worked much harder to achieve the quality status worldwide. Phcog Mag is now indexed in PubMed, SCOPUS, ISI Web of knowledge and many other indexing agencies.

Obtaining indexing with PubMed was a tremendous undertaking. From the authors’ point of view there is a higher priority to publish papers in journals indexed in PubMed. Publishing in a journal that is not indexed in PubMed is not as desirable because the journal receives less exposure. The Phcog Mag can now offer greater exposure of articles through inclusion in PubMed. Obtaining indexing in PubMed is indeed rewarding and quite an accomplishment.

In addition to additional indexing, we have entirely evolved the manuscript processing and peer review into an online system via www.journalonweb.com/pm. This speeds up manuscript processing, reduces costs and is a better experience for our authors and reviewers. We have received numerous positive comments on this improved electronic system, and we are indebted to our publisher, Medknow Publications and Media Pvt. Ltd, Mumbai.

The major changes in editorial policy that are to be effective from Jan 2011 are:

- Editorial process will be more transparent.
- Basic studies dealing with extracts and preliminary pharmacological/phytochemical studies will no longer be published.
- Increase visibility of insightful papers across the broad readership by highlighting them in a new front section.
- Including more newer sections in journal content.
- Abstracts to be published in structured format.
- References included in the manuscript to be furnished along with DOI numbers.

Each issue covers different topics in natural product drug discovery, and also publishes manuscripts that describe investigations, clinical reports, methods, techniques and applications of all forms of medicinal plant research and that are of broad readership interest to users in industry, academia and government. Phcog Mag is a must-read journal for medicinal plant researchers.

The journal is being indexed with CAB Abstracts, Caspur, Chemical Abstracts, CSA databases, DOAJ, EBSCO Publishing’s Electronic Databases, Excerpta Medica/EMBASE, Google Scholar, Hinari, Index Copernicus, Indian Science Abstracts, Journal Citation Reports, OpenJ-Gate, PrimoCentral, ProQuest, PubMed, Pubmed Central, Science Citation Index Expanded, SCOLOAR, SCOPUS, SIIC databases, Summon by Serial Solutions, Ulrich’s International Periodical Directory and Web of Science.

Phcog Mag. made us possible to expand many journals viz., Pharmacognosy Research [www.phcogres.com], Pharmacognosy Reviews [www.phcogrev.com], Pharmacognosy Journal [www.phcogj.com], Journal of Natural Pharmaceuticals [www.jnatpharm.org], Unani Research [www.unanires.org], etc.

Present days have seen many journals releasing every alternate day in the field of Pharmacy and natural products; Phcog Mag has become unbeatable and one of most reputed publication in terms of quality. Presently, it needs to be strengthened financially to support our continuing efforts in expanding quality journals. Author subscription is now made mandatory for all accepted articles to be made effective for papers publishing from Jan 2011.

Phcog Mag will continue publishing special sections that are of current topical interest to our readers. We welcome ideas for special sections, especially for topics that span a broad range in medicinal plants research. The new editorial team is excited and honored by the tasks and challenges ahead. We welcome the opportunity to contribute to the future of medicinal plants and to advance the field and its applications. We hope that many of you will join us as authors and reviewers in this process.

